# Induction of cell-cell fusion by ectromelia virus is not inhibited by its fusion inhibitory complex

**DOI:** 10.1186/1743-422X-6-151

**Published:** 2009-09-29

**Authors:** Noam Erez, Nir Paran, Galia Maik-Rachline, Boaz Politi, Tomer Israely, Paula Schnider, Pinhas Fuchs, Sharon Melamed, Shlomo Lustig

**Affiliations:** 1 Department of Infectious Diseases, Israel Institute for Biological Research, Ness-Ziona, Israel; 2 Department of Biology, Viral Immunology Center, Georgia State University, POB4118, Atlanta, GA 30302, USA

## Abstract

**Background:**

Ectromelia virus, a member of the Orthopox genus, is the causative agent of the highly infectious mousepox disease. Previous studies have shown that different poxviruses induce cell-cell fusion which is manifested by the formation of multinucleated-giant cells (polykaryocytes). This phenomenon has been widely studied with vaccinia virus in conditions which require artificial acidification of the medium.

**Results:**

We show that Ectromelia virus induces cell-cell fusion under neutral pH conditions and requires the presence of a sufficient amount of viral particles on the plasma membrane of infected cells. This could be achieved by infection with a replicating virus and its propagation in infected cells (fusion "from within") or by infection with a high amount of virus particles per cell (fusion "from without"). Inhibition of virus maturation or inhibition of virus transport on microtubules towards the plasma membrane resulted in a complete inhibition of syncytia formation. We show that in contrast to vaccinia virus, Ectromelia virus induces cell-cell fusion irrespectively of its hemagglutination properties and cell-surface expression of the orthologs of the fusion inhibitory complex, A56 and K2. Additionally, cell-cell fusion was also detected in mice lungs following lethal respiratory infection.

**Conclusion:**

Ectromelia virus induces spontaneous cell-cell fusion in-vitro and in-vivo although expressing an A56/K2 fusion inhibitory complex. This syncytia formation property cannot be attributed to the 37 amino acid deletion in ECTV A56.

## Background

Orthopox viruses are a family of large DNA viruses that replicate in the cytoplasm of infected cells. There are two major infective forms of the virus: a single-membrane wrapped virion also known as mature virion (MV) and a double-membrane wrapped virion, also known as enveloped virion (EV) [1]. An additional subdivision is used to describe the different intracellular and extracellular forms of the virus. The intracellular progeny is subdivided to a single-membrane wrapped virion also named as intracellular-mature-virus (IMV) and to intracellular-enveloped-virus (IEV) which is wrapped with two additional membranes. The extracellular forms are divided to an extracellular-cell-associated-virus and to the extracellular-enveloped-virus (CEV and EEV respectively) [2]. Attachment of EV particle to the cell results in the rupture of the outer membrane by glucose-amino glycans (GAGs) revealing single-membrane wrapped particle: the MV. At this stage the mechanism, of entry is identical to that of naked MV particle. During MV entry, the membrane fuses either with the host-cell plasma membrane or with the endosome membrane, releasing the viral core into the cytoplasm [3]. Previous studies with the orthopox prototype vaccinia virus (VACV) or cowpox (CPXV) virus showed that artificial decrease of the medium pH results in the fusion of virus infected cells and syncytia formation. Syncytia formation under low-pH conditions is largely separated into two major routes: One is induced by large number of viral particles which are present in the medium, attach the cell membrane and thus induce fusion "from without". The other results from high amount of intracellular viral particles, which induce fusion "from within" [1].

Recently, a group of viral proteins was characterized as the entry-fusion-complex (EFC). This complex comprises at least 8 viral proteins: A16, A21, A28, G3, G9, H2, J5 and L5 [4]. It was shown that deletion of certain members of this complex result in inhibition of virus entry and of pH-dependent cell-cell fusion. Thus, the current model for poxvirus-induced cell-cell fusion relates syncytia formation to viral entry [1]. Early studies of the poxvirus hemagglutinin showed that hemagglutinating strains such as vaccinia strain Western Reserve (VACV-WR), VACV-IHD-J and CPXV do not induce syncytia at neutral pH conditions, whereas at the same conditions, strains that do not exhibit hemagglutinating properties (VACV-IHD-W, rabbitpox) induce cell-cell fusion [5]. Later it was demonstrated that deletion of the hemagglutinin gene, namely A56R, or inhibition of its protein product by inhibitory antibodies result in the formation of syncytia by the strains mentioned above under neutral pH conditions. In addition, K2, a serine protease inhibitor (SPI-3) was also shown to play a role in the fusion process [6]. Later on, K2 was shown to form a complex with A56R in infected cells and addition of anti K2 antibodies to the medium of CPXV infected cells also results in cell-cell fusion under neutral pH conditions [7]. Thus, it is believed that the A56 and K2 form a complex which is inhibitory to syncytia formation in poxviruses [1].

In this study we describe the formation of syncytia by another member of the orthopox family, namely ectromelia virus (ECTV) which is the causative agent of the mousepox disease in mice [8]. We show that ECTV induces syncytia formation under neutral pH conditions and in the lungs of infected mice. This cell-cell fusion process requires infection at high multiplicity of infection (MOI) or following infection, replication and maturation of the virus. We show that inhibition of virus maturation or migration to the cell membrane inhibits cell-cell fusion, whereas inhibition of virus egress or neutralization of extracellular particles does not affect syncytia formation. We further show that cell-cell fusion occurs despite cell surface expression of the fusion inhibitory complex.

## Results

### ECTV induces syncytia in cultured cells

**Figure 1:**
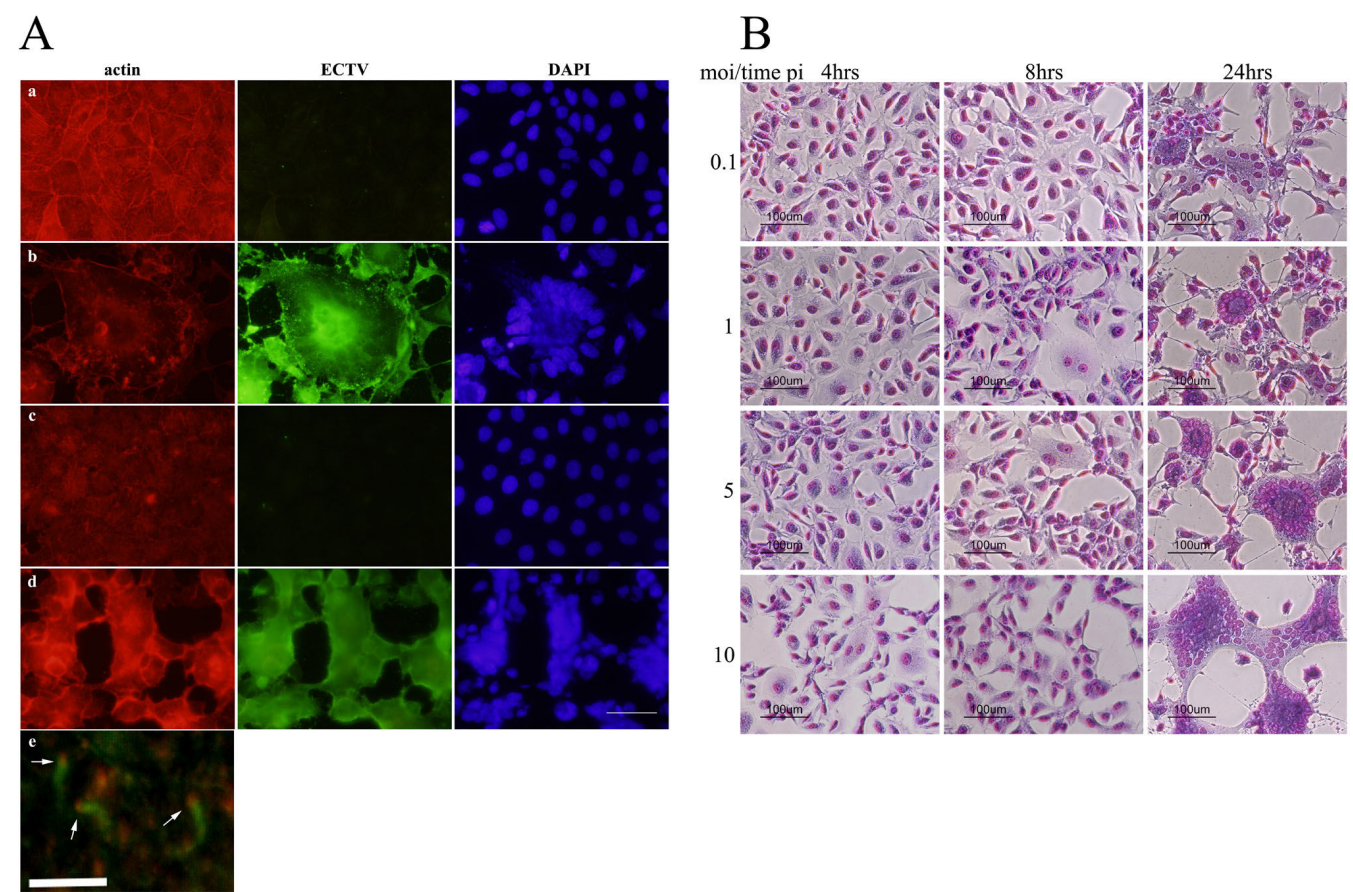
**A) Infection with ECTV induces cell-cell fusion**. BS-C-1 and HeLa cells were infected with ECTV virus at MOI = 1. 24 hrs post infection cells were fixed and stained for DAPI (blue), actin (red) and ECTV (green). BS-C-1 control (**a**), BS-C-1 infected with ECTV (**b**), HeLa control (**c**), HeLa infected with ECTV (**d**). Bar = 100 μm. **e**) Actin tails (green) with ECTV particles (red) at their tips (designated with arrows). Bar = 1 μm. **B) **Induction of syncytia by ECTV virus is time and infection titer-dependent. BS-C-1 cells were infected at different moi (0.25, 1, 5 and 10). At different time points cells were fixed and visualized by May-Grunwald and Gimsa staining. Bar = 100 μm.

In order to study its cytopathic effect in cell culture, BS-C-1 cells were infected with ECTV at MOI = 1. The cells were incubated at pH 7.4 and 24 hours post infection (hpi) cells were fixed, stained and cytopathic effect was evaluated by immunofluorescence microscopy. The most prominent cytopathic effect by ECTV was the formation of syncytia which was manifested by polykaryocytosis (Fig. 1Aa and 1Ab). These giant multi-nucleated cells comprised few to tens of nuclei which were positioned in many cases in a ring-shape form.

Importantly, formation of syncytia by ECTV occurred under neutral pH conditions and acidification of the medium did not enhance cell-cell fusion any further (data not shown). These results are in contrast to syncytia formation in VACV-WR infected cells which require acidification of the medium to around pH5.5 in order to obtain syncytia [1].

Whereas the actin cytoskeleton in un-infected control cells remained well organized and exhibited actin stress-fibers and marginal actin belt at the cell periphery (Fig 1A-a), ECTV-infected syncytia, exhibited disrupted actin cytoskeleton with no signs for actin cables and only poor actin belt is surrounding the cell (Fig. 1A-b). Additionally, infected cells contained actin-tails with ECTV at their tips (Fig. 1A-e) which are typical to pox-virus egress [9]. Syncytia formation was also observed, to a greater extent, in human cervical cells (HeLa) where polykaryocytes contained tens of nuclei and a completely disrupted actin cytoskeleton (Fig. 1A-c and 1Ad).

### Syncytia formation by ECTV is time- and dose-dependent

In order to follow the progression of syncytia formation, BS-C-1 cell were infected at different MOI and cells were fixed and stained at different time points post infection. ECTV induced syncytia in a dose- and time-dependent manner (Fig. 1B). Already 8 hours post infection cells tended to aggregate towards a center before the induction of cell-cell fusion and the formation of polykaryocytes (24 hours). In cells which were infected at MOI lower than 5, polykaryocytes appeared in a ring-shape form and the number of nuclei in multinucleated cells positively correlated with the increase of moi. Increasing the MOI to 5 or 10 further emphasized this phenomenon where polykarycytosis was demonstrated throughout the entire culture.

### Syncytia formation by ECTV requires sufficient amount of viral particles on the plasma membrane

**Figure 2:**
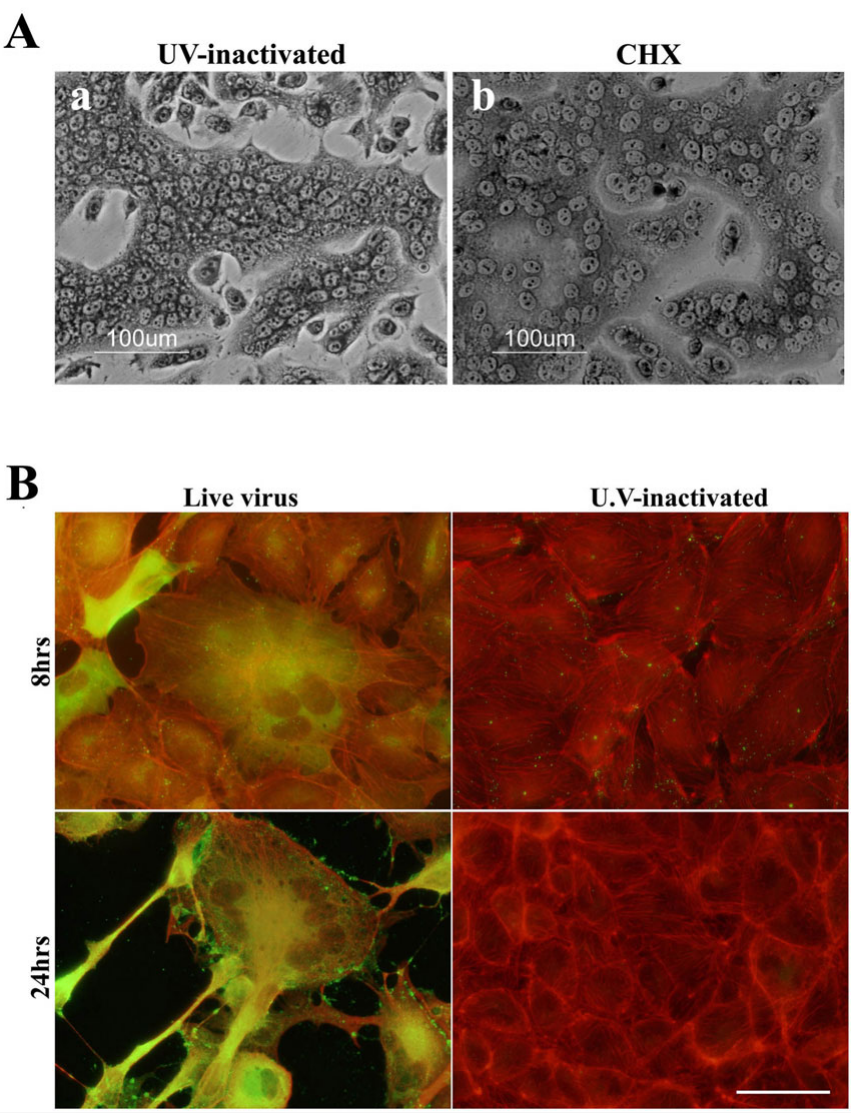
**Syncytia formation by ECTV requires sufficient amount of virus**. **A) **HeLa cells were infected with either UV-inactivated virus **(a) **or live virus in the presence of 100 μg/ml cycloheximide **(b) **at MOI = 100. The cells were fixed after 2 hours and stained with May-Grunwald and Gimsa staining. **B) **BS-C-1 cells were infected with live or U.V-irradiated virus at moi = 1. After 8 or 24 hours the cells were fixed and stained for ECTV (green) and actin (red).

Syncytia formation by poxviruses occurs by two distinct mechanisms: "from within" by intracellular virus progeny and "from without" when a large amount of virus is added to the cells. To check weather ECTV particles can induce syncytia formation also "from without" we infected HeLa cells with either "live" virus in the presence of cycloheximide (CHX, a potent inhibitor of protein synthesis) or with UV-inactivated ECTV particles at high MOI (equivalent to >100pfu/cell) at neutral pH. In both conditions, already at 2 hpi the cultured cells were fused and syncytia dominated the entire culture exhibiting giant polykaryocytes consisting tens to hundreds nuclei (Fig. 2A). In contrast, when the infection load of UV-inactivated virus was equal to MOI = 1 incubation with UV-inactivated virus did not induce cell-cell fusion. Cells maintained their normal epithelial morphology and the actin cytoskeleton was intact even at 24 hpi. No viral antigens were observed in the cytoplasm (by immunofluorescence staining) and viral particles which were apparent on the plasma membrane at 8 hpi (Fig. 2B), were already absent at later time points. Similar results were obtained in HeLa cells (not shown). In order to further address the possible role of the various infective forms of ECTV in cell-cell fusion, we inhibited cellular processes which are crucial for poxvirus morphogenesis.

**Figure 3:**
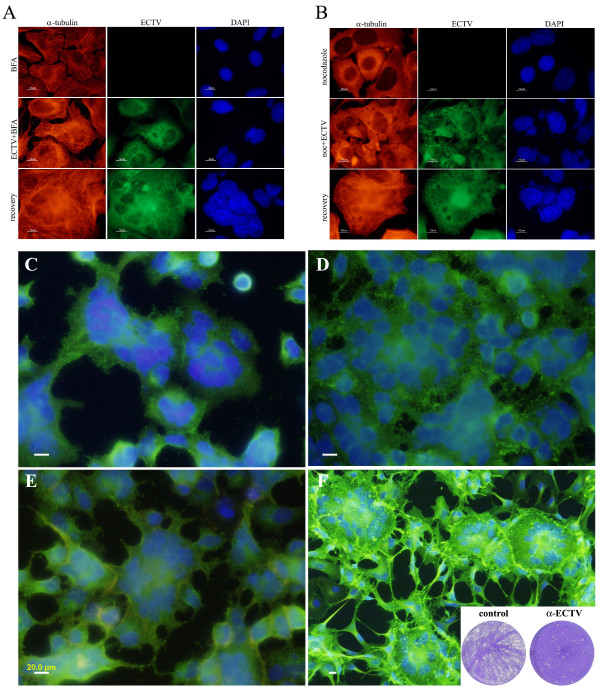
**Effect of different inhibitors on cell-cell fusion**. HeLa cells were infected with ECTV at moi = 1 and incubated for 16 hours with different inhibitors. **A) **Cells incubated with 0.5 μg/ml BFA. **B) **Cells incubated with 5 μM nocodazole. Bottom images of each panel represent cells 4 hours after withdrawal of the inhibitor. **C, D, E and F**: Cells incubated with 0.5 μM cytochalasine-D, 10 μM PP1, 10 μM STI-571 and antiserum to ECTV, respectively. Inset in **F **demonstrates ECTV comet inhibition assay with anti-ECTV serum.

**Figure 4:**
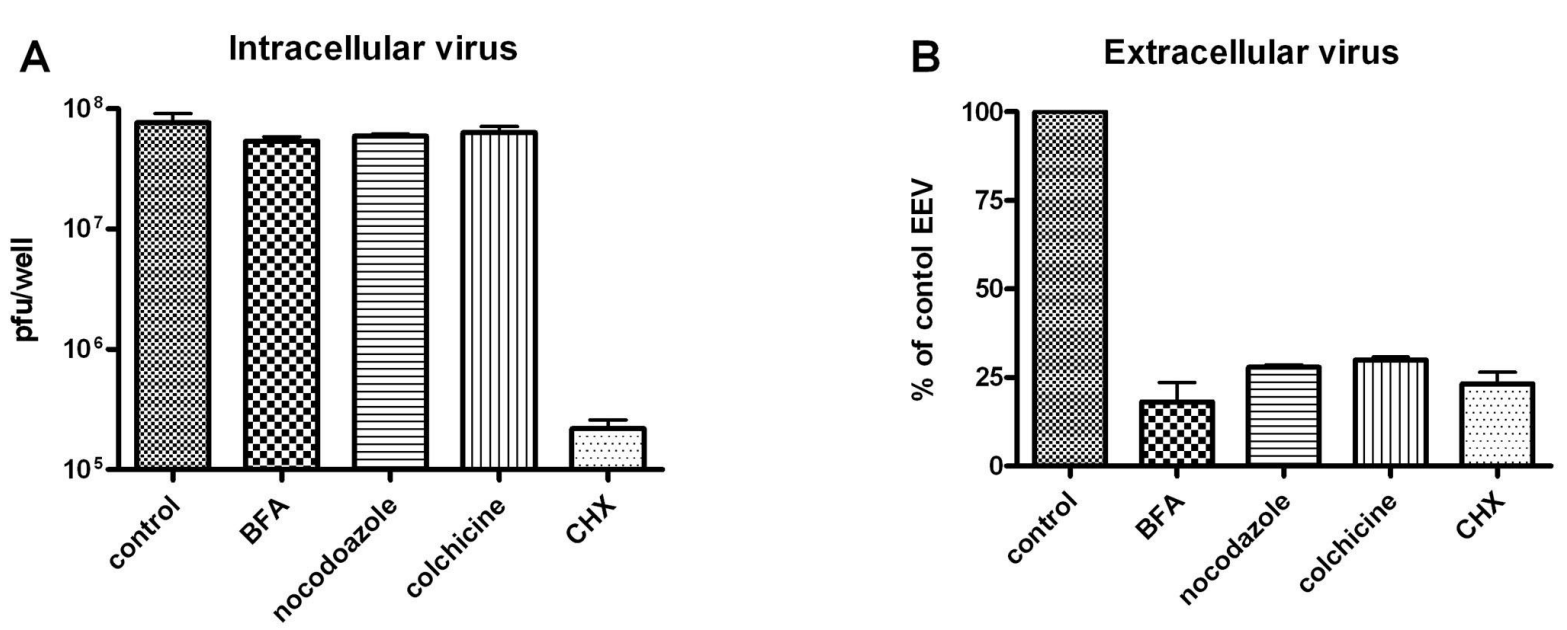
**Effect of different inhibitors on intra- and extracellular progeny of ECTV**. HeLa cells were infected with ECTV at MOI = 5 and incubated with different inhibitors (0.5 μg/ml BFA, 5 μM nocodazole, 5 μM colchicine or 100 ng/ml cycloheximide) for 24 hours. Titers of intracellular (**A**) and extracellular (**B**) virus were determined by plaque assay. Error bar = SD.

To elaborate on the role of ECTV morphogenesis in syncytia formation, we infected monolayers with ECTV at MOI = 1 and incubated the cells in the presence of 0.5 μg/ml Brefeldine-A (BFA) - a specific inhibitor of the trans-Golgi network (TGN), nocodazole, or colchicine (two specific inhibitors of microtubules dynamics) for 24 hours and followed their effect on ECTV-induced syncytia formation. Deterioration of the TGN by BFA allowed for the replication of ECTV which was manifested by positive staining in the cytoplasm (Fig. 3A) and the formation of intracellular mature infectious virions (MV) at equal levels to untreated cells (Fig. 4). However, release of infectious viral particles from the cells to the medium was inhibited by >80% (Fig 4) and syncytia formation was completely inhibited (Fig. 3A). Similar results were obtained by disruption of the microtubules network by either nocodazole (Fig 3B) or colchicine (not shown). There were no apparent changes in intracellular viral staining and intracellular virus load was not altered by either nocodazole or colchicine (Fig. 4). However, as with BFA, in cells treated with these microtubules inhibitors, extracellular virus levels dropped by 70%, staining for microtubules was diffuse and no cell-cell fusion was apparent.

In order to assure that inhibition of syncytia formation by BFA, Colchicine and Nocodazole was a direct result of morphogenesis inhibition, and not a consequence of cellular damage, we infected cells with ECTV and treated with the different inhibitors as described above. 16 hours post infection, the inhibitors were washed-out and fresh medium was added. Already 2-4 hours after withdrawal of BFA or nocodazole cell-cell fusion resumed and polykaryocytes displayed 5 nuclei or more (Fig. 3A-bottom and Fig. 3B-bottom respectively). Staining for α-tubulin demonstrates that microtubules network reformed (in the case of nocodazole- and BFA-treated cells) and that ECTV staining was all over the cytoplasm, including cell boundary. Washing out of colchicine did not resume microtubules polymerization and syncytia formation was blocked (not shown)

Poxvirus egress from the cell requires the formation of actin tails which propels cell-associated extracellular virus (CEV) away from its host cell prior to its release from the cell by enzymatic activity of the cellular kinases Src and Abl [9,10]. However, disruption of actin by cytochalasine-D (Fig. 3C) or inhibition of actin tail formation by specific src-kinase inhibitor PP1 (Fig. 3D) or SU6656 (not shown) did not inhibit ECTV-dependent cell-cell fusion. Also inhibition of virus release by the abl kinase-inhibitor STI-571 [11] did not inhibit polykaryocytes formation (Fig. 3E). Moreover, addition of inhibitory antibody to ECTV to the medium (Fig. 3F) at a concentration that inhibits comet formation (Fig. 3F, inset) did not prevent cell-cell fusion. In conclusion, the data so far points toward cell membrane associated mature virions (MV) to be involved in cell-cell fusion.

### Cell-cell fusion occurs independently of A56/K2 inhibitory complex

**Figure 5:**
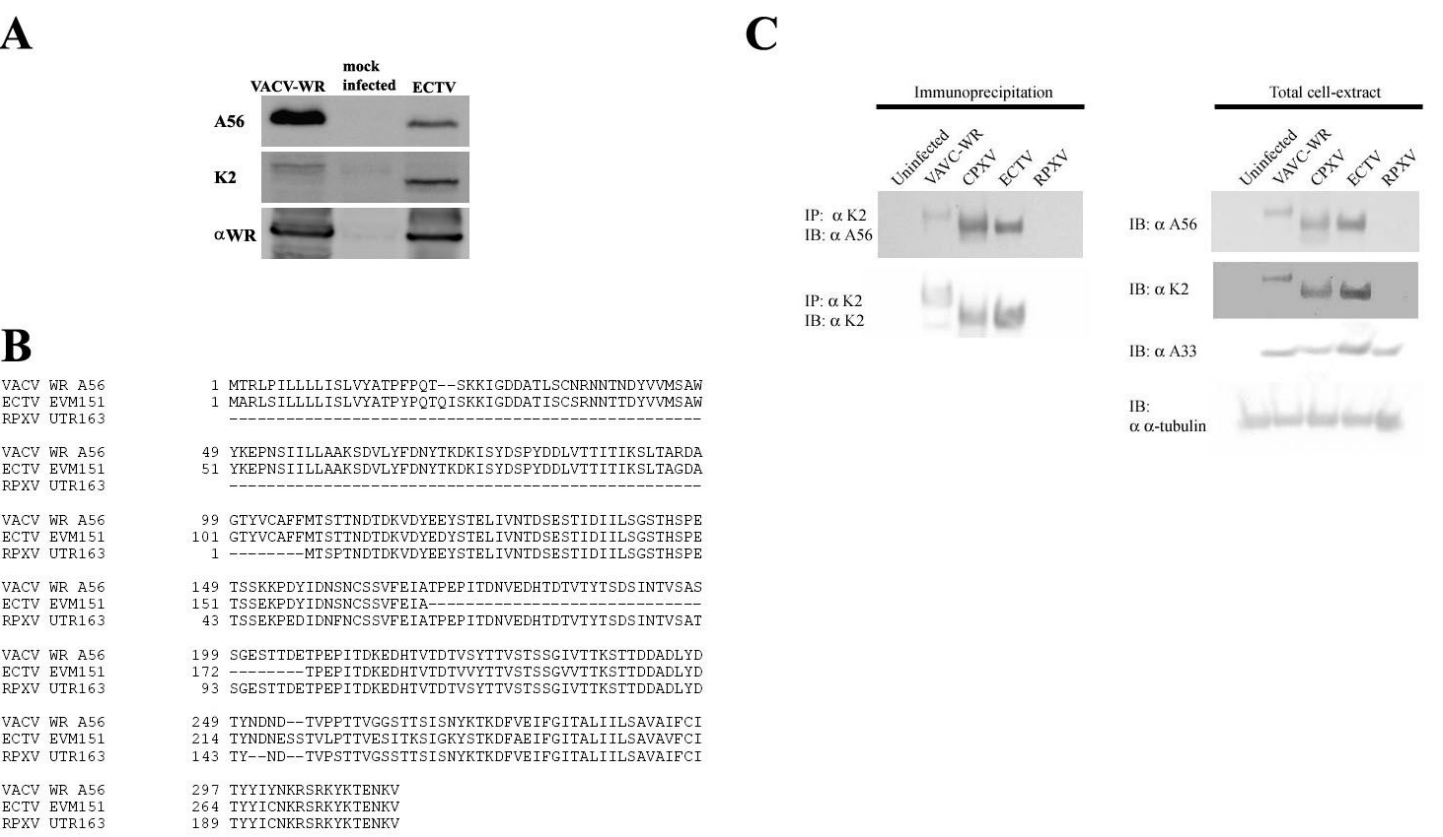
**Expression and complex formation of A56 and K2 by ECTV**. **A) **Expression of A56 and K2 by VACV-WR and ECTV. α-VACV-WR antibody was used as control to viral-proteins expression. **B) **Amino-acids sequence alignment of A56 of VACV-WR, ECTV, CPXV and RPXV. **C**) Co-immunoprecipitation of K2 and A56. Cells were infected with ECTV, VACV-WR, CPXV, RPXV or uninfected. K2 was immunoprecipitated (IP) and its interaction with A56 was evaluated by immunoblot (left side of the panel). Expression of K2, A56 as well as A33 (control for infection) and α-tubulin (cellular marker) are presented on the right side of the panel.

Early studies established the correlation between the orthopox-virus hemagglutinin (A56) and cell-cell fusion under neutral-pH conditions. Generally, poxviruses that have hemagglutination properties (i.e. VACV-WR, CPXV) induce a cytopathic effect without the formation of syncytia whereas viruses that induce cell-cell fusion (i.e. Rabbitpox, VACV-IHD-W) do not [12]. Inactivation of A56 by antibodies that inhibit hemagglutination or silencing its gene results in the formation of syncytia [5]. In addition, K2, a serine protease inhibitor, was shown to cooperate with A56 in its inhibitory effect on cell-cell fusion [6,13]. We therefore wanted to verify whether the orthologs of A56 and K2 are expressed by ECTV and to evaluate the hemagglutination properties of the ECTV A56 ortholog (EVM151). Western blot analysis showed that both orthologs of K2 and A56 were expressed in ECTV-infected cells and that both proteins have faster mobility in SDS-PAGE in comparison to their VACV-WR orthologs. (Fig. 5A). We further substantiated previous results [14] showing that the Moscow strain of ECTV, which is utilized throughout this study presents hemmaglutination ability (endpoint titer of 1:4) in comparison to the hemagglutinin-negative rabbitpox strain (RPXV) (endpoint 0) and to the hemagglutinating strain VACV-WR (endpoint 1:16).

**Figure 6:**
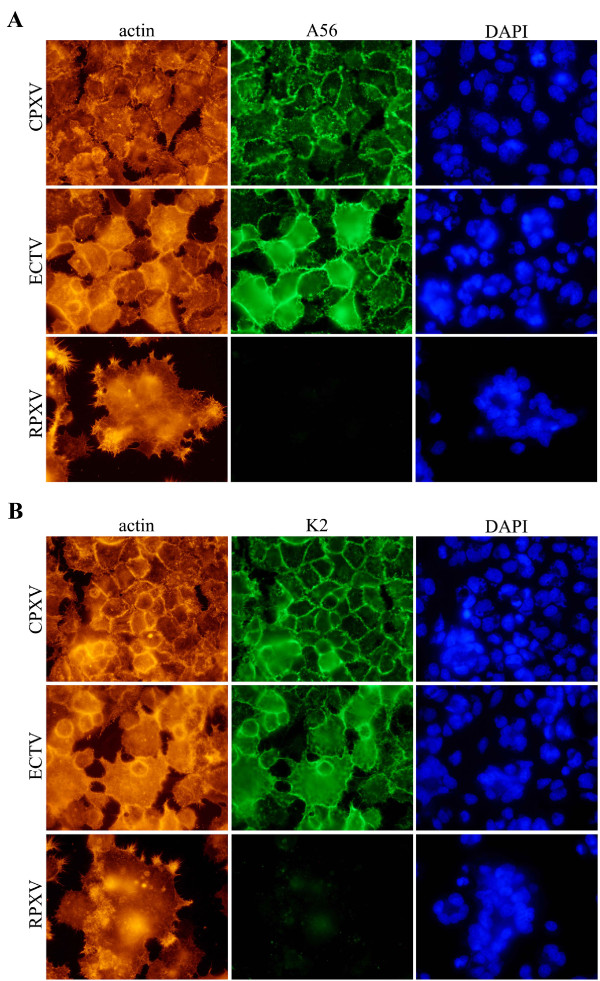
**Localization of A56 and K2 on the plasma membrane of ECTV infected cells**. HeLa Cells were infected with ECTV, CPXV or RPXV at MOI = 1. 24 hpi membrane associated A56 (**A**) or K2 (**B**) were visualized by "live" immunolabeling followed by fixation and indirect immunofluorescence staining. Actin cytoskeleton and nuclei were labeled as cellular counter-staining.

Multiple alignment of the amino-acid sequence of A56 orthologs from different poxviruses (Fig. 5B) shows that ECTV A56 ortholog is missing a 37 amino acid stretch which is present in VACV-WR and in part in Cowpox - two hemagglutinating strains. To check whether this deletion prevents interaction of A56 with K2 and by that formation of a functional inhibitory complex, we infected HeLa cells with ECTV, VACV-WR, CPXV or RPXV and verified the ability of K2 to associate with A56 by co-immunoprecipitation. Figures 5C demonstrates an interaction between A56 and K2 of ECTV, regardless of the deleted amino acid stretch, as in the case of VACV-WR and Cowpox. As K2 bares no transmembrane domain, nor a membrane anchoring motif, its presence on the plasma membrane of infected cells is mediated through its interaction with A56 [7]. This interaction forms the inhibitory fusion complex. In order to check whether a fusion inhibitory complex of the ECTV orthologs of A56-K2 is localized on the surface of infected cells, we infected cells with either ECTV, Cowpox or RPXV and 24 later immunolabelled the cells surface for either K2 or A56 by a technique that was described previously [7]. Indeed, both A56 and K2 were localized on the plasma membrane of both Cowpox and ECTV infected cells (Fig. 6A and 6B respectively). These markers were not detected in our control-RPXV-infected cells neither by immunostaining (Fig. 6) nor by immunoblotting (Fig. 5C) since K2 is rapidly secreted in the absence of its membrane anchoring counterpart, namely A56 [4]. These results demonstrate that ECTV induces syncytia under neutral pH conditions despite the presence of A56/K2 complex on the cell membrane.

**Figure 7:**
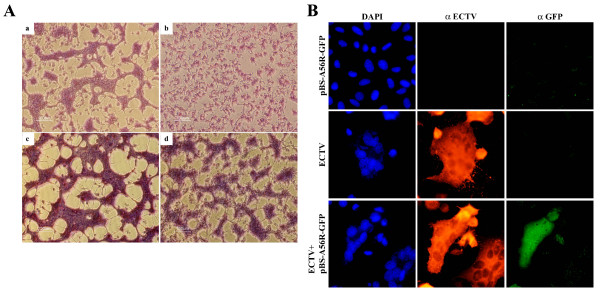
**ECTV induces syncytia regardless of A56 or K2 expression**. **A) **Co-infection of HeLa cells with ECTV and VACV-WR. Cells were infected with either ECTV (**a**) VACV-WR (**b**), or co-infected with both viruses in a ratio of 1:1 or 1:10 (**c **and **d **respectively). 24 hours post infection cells were fixed and stained. **B) **Co-transfection-infection of VACV-WR A56 and ECTV in HeLa cells. Cells were infected with ECTV at MOI = 1 and transfected with pBS-A56R-GFP as described in Materials and Moethods. 24 hpi cells were fixed and stained for GFP (green), ECTV (red) and nuclei (DAPI).

Having demonstrated that the A56 ortholog of ECTV differs in sequence and length from VACV-A56, we checked whether ectopic expression of a functional A56 with a known inhibitory effect on cell-cell fusion would inhibit syncytia formation by ECTV. For this purpose we used VACV-WR as a vector for expression of A56 which was previously shown to inhibit cell-cell fusion under neutral-pH conditions [15]. We co-infected cells with both ECTV and VACV-WR and followed their cytopathic effect. At 24 hpi VACV-WR-infected cells exhibited a typical small rounded morphology (Fig.7A-b) but did not exhibit any polykaryocytes, whereas ECTV-infected cells exhibited cell-cell fusion as described above (Fig.7A-a). Interestingly, cells that were co-infected with both ECTV and VACV-WR at MOI ratio of 1:1 or even 1(ECTV):10(WR) still formed syncytia (Fig.7A-cc and 7Ad respectively). These polykaryocytes were comparable in size and nuclei number to cells that were infected with ECTV alone.

These results were also confirmed by ectopic expression of VACV-WR A56. In order to restrict expression of VACV-WR A56 to ECTV infected cells, and to distinguish between the ECTV and VACV-WR A56 we fused the WR-A56R open reading frame to the Green Fluorescent Protein (GFP) under the regulation of the early/late p7.5 promoter. We infected HeLa cells with the ECTV at MOI = 1 followed by transfection with A56R-GFP. Expression of VACV-WR A56-GFP was detected only in ECTV infected cells. Nevertheless, this expression did not inhibit cell-cell fusion induced by ECTV infection (Fig. 7B).

### ECTV infection induces cell-cell fusion in-vivo

**Figure 8:**
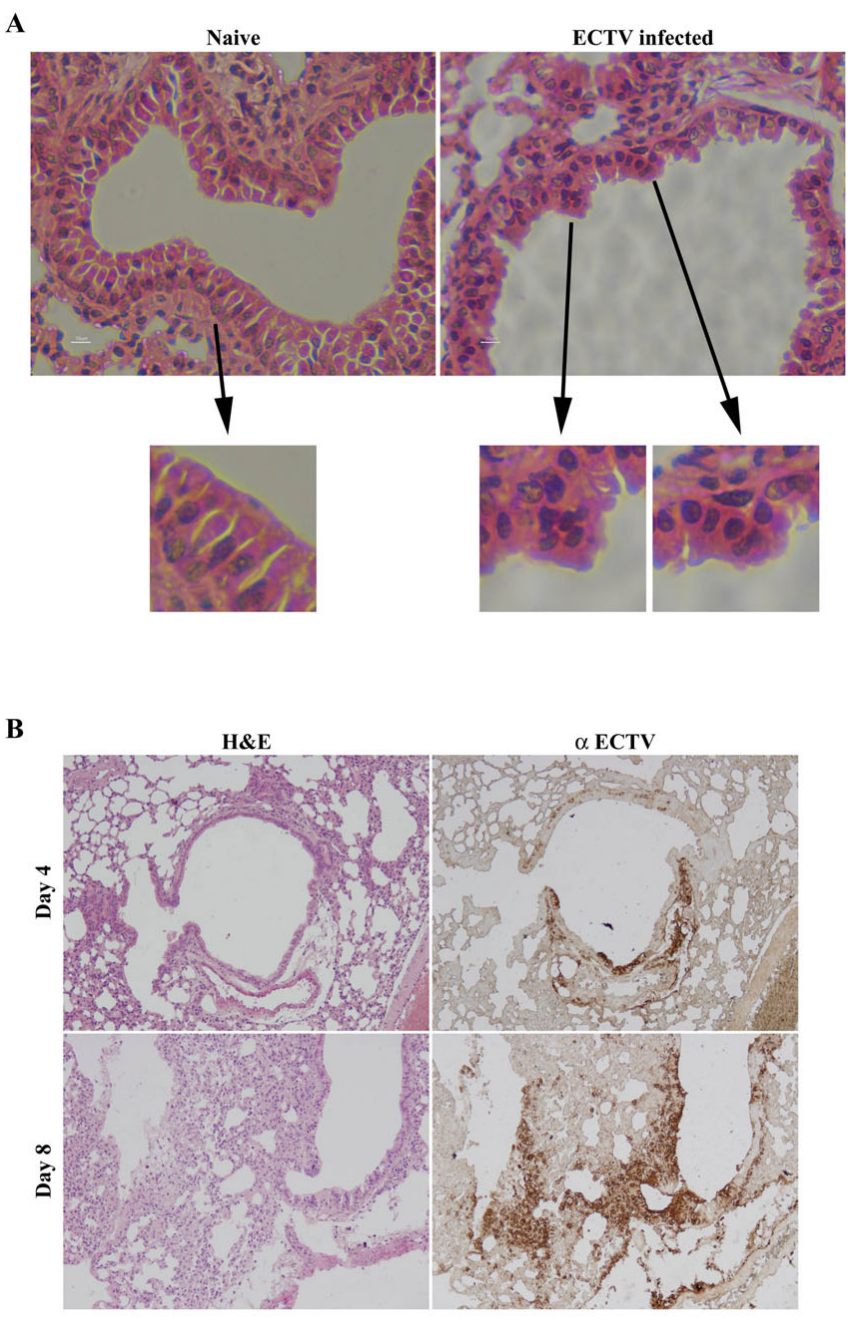
**ECTV infection induces cell-cell fusion and cellular damage in mouse lung epithelium**. BALB/c mice were infected with either mock or 10LD_50 _of ECTV by the intranasal route. At days 4-6 post infection, the animals were sacrificed and lung samples were analyzed by hematoxylin-eosine as well as immunohistochemical staining. **A) **ECTV-induced syncytia in bronchi of infected mice lung. Regions of fused cells are designated with black arrows. **B) **Damage progression in lung epithelium following ECTV infection after 4 or 8 days post infection (upper and lower images, respectively). Sequential Slices were stained by hematoxylin and Eosine (left) or labeled by immunohistochemical staining using Rabbit anti ECTV serum (right).

ECTV is a highly virulent mouse pathogen. Previous studies have shown that other poxviruses such as Monkeypox, Camelpox and Raccoonpox, which are virulent to their natural host, can induce cell-cell fusion in-vitro and in some cases also in-vivo [16-18]. Therefore, we wanted to check whether ECTV induces cell-cell fusion in-vivo. For this purpose we infected BALB/c mice with a lethal dose (10LD_50_) of ECTV by the intranasal route and examined their lungs at different time points post infection by histology. At early stages of infection (up to day 6 post infection) the lungs of ECTV infected mice kept their overall alveolar and bronchi architecture (Fig. 8A and 8B). Syncytia consisting 5 nuclei or more were also exhibited sporadically (Fig. 8A, syncytia containing and normal naive regions are designated with black arrows). At later stages, the lung epithelium exhibited progressive damage that spread to the surrounding alveoli and correlated with the presence of viral antigens as appears by immunohistochemistry (Fig. 8B).

## Discussion

Virus induced cell-cell fusion (syncytia) is a well-characterized phenomenon in many viruses [19]. In poxviruses, syncytia formation was deeply characterized by the WR strain of vaccinia virus. Induction of cell-cell fusion by VACV-WR can be achieved either directly by infection with high amount of virions per cell or several hours post infection at lower MOI, followed by virus replication and sorting to the cell membrane [1]. Under these conditions, when sufficient amount of virus is presented on the plasma membrane, brief acidification of the medium results in a fast syncytia formation. Reducing the pH is believed to resemble fusion of the viral membrane with the membrane of the acidified endosomes during virus entry or direct fusion of the viral membrane with the cell membrane [1,20]

In this study we show that ECTV induces syncytia formation in dose- and time-dependent manner. Similar to VACV, ECTV induces cell-cell fusion when sufficient amount of virus is presented on the plasma membrane; either by infection at high MOI or by allowing enough time for virus replication and sorting to the plasma membrane. However, unlike VACV which requires medium acidification for the fusion to occur, ECTV induces syncytia formation under physiological pH conditions. Infectious forms of Orthopox viruses comprise of intracellular mature viruses (MV), extracellular wrapped viruses (EV) and the intracellular enveloped viruses (IEV). These forms differ in their cellular localization and contain either single, double or three layers of membranes respectively [2]. To have an indication as to which of the different viral forms plays a role in cell-cell fusion, we utilized specific chemical inhibitors to several cellular processes aiming to block poxvirus maturation at different stages. Brefeldin-A (BFA) is a specific inhibitor which disrupts the trans-Golgi network (TGN) and hence inhibits wrapping of the virus with the two additional membranes [21] and thereby prevents the formation of IEV. The transport of IEV towards the cell membrane is facilitated by microtubules dynamics [22,23]. Therefore, perturbation of microtubules dynamics prevents microtubules-dependent, intracellular transport of poxviruses. Using these inhibitors, we showed that when virus egress is inhibited, cell-cell fusion is prevented. It is worth mentioning that these inhibitors did not affect production of infectious intracellular mature virions (Fig. 4). When later stages of virus release were inhibited by inhibitors, such as PP1 which inhibits actin tail formation or STI-571 which inhibits virus release from the plasma membrane, or by inhibiting extracellular virions using neutralizing antibodies (Fig. 3), cell-cell fusion still occurred. These results point towards the cell-membrane- associated-mature virion (MV) as the viral particle which probably mediates cell-cell fusion.

Previous studies have identified a fusion entry complex embedded within the membrane of the mature virion (MV) [1] and an inhibitory fusion complex which comprises of two viral proteins: the poxvirus hemagglutinin-A56 and the serine protease inhibitor 3 (SPI-3) K2 which is presented on the plasma membrane of poxvirus infected cells [13,24,25]. Classical studies with poxviruses have established a counter relationship between hemagglutination and fusion properties of the poxviruses. Hence, poxviruses which hemagglutinate red blood cells, do not induce spontaneous cell-cell fusion, and vice-versa [5]. Deletion of either genes or inhibition of their protein products by inhibitory antibodies results in syncytia formation [5,15,26]. Recent studies clearly demonstrated that the presence of A56 and K2, whether expressed by the virus, or expressed by the cellular machinery, inhibit cell- cell fusion [25].

In this article we show that ECTV, which is a hemagglutinating strain, expresses the orthologs of the two proteins, EVM151 (ortholog of VACV-A56) and EVM23 (ortholog of K2) (Fig. 5A). Thus, cell-cell fusion is induced by ECTV regardless of the expression of these two proteins. According to SDS-PAGE analysis, the two proteins have faster mobility than their orthologues from VACV-WR. This could be explained by the 37 amino acids deletion in EVM151 (Fig. 5B) and by differences in glycosylation pattern of EVM23 as predicted by its amino acids sequence (data not shown).

The fact that EVM151 bares a 37 amino acids deletion raised the possibility that this deletion might be responsible for the fusogenic property of the virus. Previous studies have identified different mutants of VACV which induce spontaneous cell-cell fusion while retaining hemagglutination properties [27]. However, none of these mutations resides in the 37 amino acids stretch which is deleted in ECTV hemagglutinin. We wanted to exclude the possibility that this deletion prevents interaction of A56 with K2 and thus fusion inhibitory complex cannot form. Indeed, co-immunoprecipitation assay showed that EVM23 interacts with EVM151 in a similar manner to this interaction in VACV-WR or cowpox virus. Additionally, we showed that K2 and A56 are localized properly on the plasma membrane of ECTV infected cells (Fig. 6).

We also showed that expression of A56 from a hemagglutinating strain (VACV-WR) in ECTV infected cells could not inhibit cell-cell fusion (Fig. 7B). This was further substantiated by co-infection of ECTV and VACV-WR. In this experiment cells infected by both viruses expressed a functional A56/K2 inhibitory complex. However, the appearance of syncytia indicates that ECTV-dependent cell-cell fusion is not a consequence of an un-functional inhibitory complex.

Recent studies have established a model for poxvirus entry. In this model, the poxvirus entry fusion complex (EFC) comprises at least 8 proteins: H2, G3, A28, A21, L5, J5, G9 and A16. Each of these proteins is crucial for the formation of an active EFC. Within the EFC, G9 and A16 form a complex which interacts with the fusion inhibitory - A56/K2 - complex which is presented on the plasma membrane of poxvirus-infected cells[6,13]. This interaction prevents the fusion of the intracellular mature virus membrane with the plasma membrane of an already infected cell [13]. When either A56 or K2 are inactive, the inhibitory interaction between the EFC and the A56/K2 complex is abrogated and hence cell-cell fusion is apparent (i.e. RPXV). In this study we demonstrated that ECTV expresses and presents the A56/K2 complex on the plasma membrane of infected cells yet cell-cell fusion still occurs, suggesting that in ECTV the EFC is no longer inhibited by the A56/K2 complex. Since A56 and K2 do not seem to be the cause for this lack of interaction, the reason might reside in the structure/function of either G9 or A16. The amino acids compositions of these two proteins were compared to their orthologs in VACV but only minor changes in their sequence were found. These minor changes might affect the interaction of the EFC with the A56/K2 complex even though we cannot exclude the role of other possible members of the EFC in cell-cell fusion.

Induction of cell-cell fusion under neutral pH conditions have been documented with other virulent poxviruses such as Monkeypox [17], Raccoonpox and Volepox [28] We showed here that syncytia formation in ECTV infected cells can be detected in lung epithelium following lethal respiratory infection of mice, the natural host of ECTV. Whether a similar mechanism of cell-cell fusion exists in other natural Orthopox viruses is yet to be investigated.

## Methods

### Cells and viruses

BS-C-1 (ATCC, CCL-26) and HeLa (ATCC, CCL-2) cells were routinely maintained in Dulbecco's Modified Eagle Medium (DMEM) supplemented with 10% fetal calf serum, 2 mM glutamine, 0.1 mg/ml streptomycin, 100 units/ml penicillin, 1.25 units/ml nystatin and non-essential amino acids (Biological Industries, Israel). VACV-WR (ATCC VR-119), VACV-IHD-J, RPXV (strain Utrecht, ATCC VR-157), CPXV (strain Brighton, ATCC VR-302) and ECTV (strain Moscow, ATCC VR-1374), were grown in HeLa cells and purified as described previously [29]. Viral titers were determined by plaque assay on BS-C-1 monolayers.

For inactivation of ECTV, purified virus (10^8^pfu/ml) in PBS was UV-irradiated with a PHILIPS G30T8 sterilamp in open 60 mm dish for 15 minutes. The virus suspension was gently agitated during the treatment and virus inactivation was validated by plaque assay.

### Hemagglutination assay

Hemagglutination properties of VACV-WR, RPXV and ECTV were preformed on 10^8^pfu/ml virus stocks and evaluated as described elsewhere [30].

### Antibodies and reagents

For production of Rabbit anti- IMV and anti EEV antisera - Vaccinia IHD-J was propagated in a suspension of HeLa S3 cells grown in DMEM supplemented with 2% FCS. Cells were infected at an MOI of 0.1 and when CPE was evident the medium was clarified from cell debris by centrifugation (200 g). Intracellular progeny was purified from the cells by three freeze-thaw rounds followed by sonication (3 times 4,000 Joules 1 minute each). The resulting suspension was separated from cell debris by low speed centrifugation (200 g, 5 minutes at 4°C) loaded on a 36% (W/V) sucrose cushion and concentrated by ultracentrifugation (13,500 RPM, SW28 Beckman rotor) at 4°C for 80 minutes. The resulting pellet was suspended in PBS and purified through a sucrose gradient (25-50% W/V) by centrifugation at 40000 RPM with Ti 45 rotor (Sorval/Beckman) at 4°C for 80 minutes.

Extracellular virus particles were purified from the culture media by ultracentrifugation as described above. The pellet was re-suspended in PBS and purified through a sucrose gradient (25-50% W/V) by ultracentrifugation. The bands of IMV and EEV particles were dialyzed against PBS, titrated on BS-C-1 monolayers and inactivated by betapropiolactone (βPL) (Serva, Germany).

For preparation of the anti IMV and anti EEV antisera, rabbits (New Zealand white, female) were vaccinated subcutaneously with equivalent dose of 1 × 10^6^pfu of the desired antigen (βPL inactivated IMV or EEV) formulated in complete Freud adjuvant (Sigma) followed by 3 additional injections every 21 days with the same antigen formulated in incomplete Freud adjuvant.

For production of anti Ectromelia hyper immune sera, rabbits (New Zealand white, female) were vaccinated subcutaneously with 1 × 10^7^pfu, 4 times every 21 days with viable, sucrose purified virus in PBS. Blood was collected 14 days after the final boost and separated in collection tubes (BD) by centrifugation.

Anti A33 antiserum was produced by vaccination of rabbits (New Zealand white, female) with bacterial expressed, purified His-tagged A33 formulated with complete Freud adjuvant followed by 2 additional injections every 21 days with the same antigen formulated in incomplete Freud adjuvant.

Rabbit anti K2 and Rabbit anti A56 were a kind gift from Dr. Bernard Moss (NIH, USA) [4]. Mouse anti HA (clone 1H831) was kindly provided by Dr. Hisatoshi Shida (Hokkaido University, Japan), Mouse anti SPI-3 (clone 4A11-4A3) was kindly provided by Dr. Richard Moyer (UFL, USA).

Mouse anti α-tubulin was purchased from Sigma immunochemical (MO, USA). As secondary antibody reagents, we used Alexa-555-conjugated goat anti mouse or anti rabbit antibodies and Alexa488-conjugated goat anti mouse or goat anti rabbit antibodies (Invitrogen, USA). For Western-blot we used HRP-conjugated goat anti rabbit antibody (Roche). Actin filaments were labeled with TRITC or FITC-labeled Phalloidin and nuclei were visualized by 4',6-Diamidino-2-phenylindole (DAPI), all from Sigma Immunochemicals. The following inhibitors: Brefeldine-A (BFA), cycloheximide, Arabinoside-cytosine (Ara-C), PP1, nocodazole, colchicine and cytochalasin-D were also from Sigma Inmmunochemicals.

### Infection and comet assay

For infection with the different poxviruses, manslayers of cells in 6-well or 12-well dish were rinsed twice with PBS before virus, at the desired MOI, was added to the medium in a volume of 0.2 ml. The cells were incubated at 37°C or 4°C for one hour, rinsed with PBS and further incubated in Eagle's minimal essential medium supplemented with 2% FCS at 37°C.

Comet assay was preformed as described elsewhere [31].

#### Immunofluorescence staining, histology, immunohistochemistry and image acquisition

For Immunofluorescence labeling cells were plated on 13 mm round glass coverslips (Marienfeld, Germay). Following the different infections and treatments cells were fixed in 3% paraformaldehyde (PFA) in PBS for 20 minutes and permeabilized with 0.5% Triton X-100 for 2 minutes. The fixed cells were rinsed with PBS, blocked with 2% Bovine Serum Albumin (Sigma) and the coverslips were incubated for 45 minutes with the primary antibodies. After washing with PBS, cells were incubated with the appropriate fluorescent secondary antibodies for 30 minutes. For DNA visualization we incubated the coverslips with 5 μg/ml DAPI. After additional washes with PBS, the coverslips were mounted with Elvanol (Mowiol, 4-88, Hoechst, Germany) on glass slides.

Histology was preformed on thin paraffin-embedded sections. Serial sections were stained with either hematoxylin and eosin or stained with anti-VACV hyper-immune serum using EnVision kit (Dakocytomation).

Microscopy and Fluorescence microscopy were carried out using the Nikon TE2000 fluorescent microscope. The images were acquired by a Nikon DXM-1200F camera and processed with Adobe Photoshop software.

### One step growth

Cells were infected with ECTV at MOI = 5 for 1 hour at 37°C as described above. 2 hours post infection different chemical inhibitors were added at the desired concentrations and the cells were further incubated at 37°C for 24 hours. At this time point the medium was gently collected from the dishes and cell debris were separated by centrifugation. Cells were scrapped, underwent freeze-thaw steps for 3 times and sonicated 3 times for 30 seconds. Intracellular and extracellular viral titers were determined by plaque assay on BS-C-1 monolayers.

### Plasmids and co-transfection-infection

Construction of VACV-WR A56R fused to GFP under the regulation of p7.5 early/late promoter was carried out by the polymerase chain reaction (PCR).

For the p7.5 promoter we used the VACV genome as a template and the primers NE5: AA**CTGCAG**TTTTTATAGTAAGTTTTT (PstI site in bold) and NE16: TCCC**CCCGGG**CTACTTCCTTACCGTGCA (XmaI site in bold). For the A56R gene of VACV-WR we used the primers NE17: TCC**CCCCGGG**ATGACACGATTACCAATA (XmaI site in bold) and and NE18: CGC**GGATCC**GACTTTGTTCTCTGTTTT (BamHI in bold).

The GFP gene was amplified using the primers NE20: CGC**GGATCC**ATGGTGAGCAAGGGCGAG (BamHI site in bold) and NE21: TTTTCCTTTT**GCGGCCGC**TTACTTGTACAGCTCGTC (NotI site in bold).

The resulted DNA fragments were digested by restriction enzymes acroding to their restriction sites and ligated with T_4 _ligase. The resulting p7.5-A56R-GFP fragment was amplified by PCR using primers NE5 and NE21 and the PCR product was inserted into PstI and NotI sites of pBluescript vector (Stratagene) to give pBS-A56R-GFP.

For co- transfection-infection experiments, HeLa cells were grown on 13 mm coverslips in 24 well dish until reaching 80-90% confluence. Cells were infected with ECTV at MOI = 1 for 1 hr at 37°C, washed twice with DMEM and transfected with 2 μg pBS-A56R-GFP by Lipofectamine 2000 (invitrogen). 24 hours later cells were fixed and stained for the desired proteins.

### Immunoblot analysis

HeLa cells were infected with VACV-WR, CPXV, RPXV or ECTV at MOI = 1. After 1 hour, the cells were washed three times with PBS and further incubated at 37°C. 24 hpi the dishes were washed twice with PBS and the cells were scrapped with rubber policeman. Immunoprecipitation, SDS-page and Western-blot analysis were preformed as described previously [32].

## Competing interests

The authors declare that they have no competing interests.

## Authors' contributions

NE carried out the experiments and data analysis, cloned the pBS-A56R-GFP plasmid for transfections and drafted the manuscript. NP and SL participated in experiments design, data analysis and manuscript preparation. GMR did the immunoprecipitation and immunoblot assays. BP participated in in-vivo experiments and prepared histology samples. PS and PF participated in cell-culture infections. TI and SM participated in in-vivo experiments.
